# Plant neighbor identity influences plant biochemistry and physiology related to defense

**DOI:** 10.1186/1471-2229-10-115

**Published:** 2010-06-17

**Authors:** Amanda K Broz, Corey D Broeckling, Clelia De-la-Peña, Matthew R Lewis, Erick Greene, Ragan M Callaway, Lloyd W Sumner, Jorge M Vivanco

**Affiliations:** 1 Department of Horticulture and Landscape Architecture and Center for Rhizosphere Biology, Colorado State University, Fort Collins, CO, 80523, USA; 2 Proteomics and Metabolomics Facility, Colorado State University, Fort Collins, CO, 80523, USA; 3 Division of Biological Sciences, University of Montana, Missoula, MT 59812, USA; 4 The Samuel Roberts Noble Foundation, Plant Biology, 2510 Sam Noble Parkway, Ardmore, OK 73401, USA

## Abstract

**Background:**

Chemical and biological processes dictate an individual organism's ability to recognize and respond to other organisms. A small but growing body of evidence suggests that plants may be capable of recognizing and responding to neighboring plants in a species specific fashion. Here we tested whether or not individuals of the invasive exotic weed, *Centaurea maculosa*, would modulate their defensive strategy in response to different plant neighbors.

**Results:**

In the greenhouse, *C. maculosa *individuals were paired with either conspecific (*C. maculosa*) or heterospecific (*Festuca idahoensis*) plant neighbors and elicited with the plant defense signaling molecule methyl jasmonate to mimic insect herbivory. We found that elicited *C. maculosa *plants grown with conspecific neighbors exhibited increased levels of total phenolics, whereas those grown with heterospecific neighbors allocated more resources towards growth. To further investigate these results in the field, we conducted a metabolomics analysis to explore chemical differences between individuals of *C. maculosa *growing in naturally occurring conspecific and heterospecific field stands. Similar to the greenhouse results, *C. maculosa *individuals accumulated higher levels of defense-related secondary metabolites and lower levels of primary metabolites when growing in conspecific versus heterospecific field stands. Leaf herbivory was similar in both stand types; however, a separate field study positively correlated specialist herbivore load with higher densities of *C. maculosa *conspecifics.

**Conclusions:**

Our results suggest that an individual *C. maculosa *plant can change its defensive strategy based on the identity of its plant neighbors. This is likely to have important consequences for individual and community success.

## Background

In microbial communities, the perception of conspecific (same species) neighbors by an individual bacterium can elicit specific biochemical and behavioral responses that are required for bacterial virulence [1,2]. Alternatively, perception of heterospecific (different species) bacterial neighbors or even different strains of the same bacterial species can lead to entirely different, often antagonistic responses [1,2]. In a similar way, social insects such as ants are able to recognize and differentially respond to colony members versus colony invaders [3]. This recognition is modulated by chemical and biological signals that impact the fitness of individual ants and the success of the colony as a whole [3,4]. Perception and recognition of conspecifics by mammals often requires multiple chemical, biological and behavioral cues. These processes affect mate recognition, antagonism, and immune response, impacting individual fitness [5,6]. Thus, organisms ranging from the simplest to the most complex differentially perceive and respond to conspecific and heterospecific neighbors. Although this type of neighbor perception and response system is widely recognized in other taxa, it has to a large extent been neglected in studies of plants (but see [7-11]).

It is generally accepted that plants are able to recognize and respond to multiple biotic and abiotic stimuli. For instance, changes in the availability of nutrients and light prompt both morphological and chemical changes within the plant [12,13], which are often associated with specific changes in gene expression [14]. Similarly, plants exhibit both general and specific responses to a variety of pathogens, mutualists and herbivores at the biological, molecular and chemical levels [15-18]. However, the ability of an individual plant to differentially recognize and respond to neighboring plants remains a subject of debate [11,19-21]. The ways in which plants recognize and respond to all aspects of their environment will influence their competitive ability in a given ecosystem, and can thus have important consequences for the overall success of a species.

Competition between organisms is thought to be largely responsible for structuring ecological communities and may help to explain observed spatial patterns of species density and diversity. Due to the sessile nature of plants, spatial distribution greatly influences the amount of conspecific and heterospecific competition an individual plant experiences. The spatial distribution of plants in a community impacts the way in which plants interact with each other, with mutualists [22], with consumers [23] and with other aspects of the environment. Competition between conspecifics can be strikingly different than competition between heterospecific plant neighbors [24], and plant neighbor identity can alter plant growth habits, metabolism [25] and gene expression [26].

In experimental communities, high relative densities of conspecifics affect parameters such as growth, fecundity [27] and the production of defense compounds [28,29]. In addition, high relative densities of conspecifics can suppress competitively superior species [27], enhance facilitative relationships [22], and increase herbivore loads [30]. However, the impact of conspecific density on species performance is expected to differ depending on the plant species and its competitive competency [27].

Exotic invasive plants would appear to have substantial competitive competency, as they are often observed to displace native species and are typically considered a major threat to biodiversity in native ecosystems [31]. Invasive plants often establish very high relative population densities, resulting in a near monoculture of conspecific neighbors. However, they also exist at much lower relative densities within in a matrix of heterospecific neighbors. Because of this wide variation in relative density, invasive plants represent useful ecological models with which to examine the effect of plant community composition on plant biochemistry under natural field conditions. Biochemical characteristics of plants may play a role in invasive success [32,33], and thus a detailed understanding of plant biochemistry is likely to provide insight into the mechanisms of invasion [34].

*Centaurea maculosa *Lam. (*Centaurea stoebe *L. spp. *micranthos *(Gugler) Hayek, *C. biebersteinii*, spotted knapweed) is a particularly problematic invasive weed in the north western United States that tends to form dense stands, displacing native species. We were interested in determining if an individual *C. maculosa *plant would alter its defensive strategy due to the presence of conspecific versus heterospecific plant neighbors. In an initial greenhouse experiment we manipulated plant neighbor identity (conspecific: *C. maculosa *or heterospecific: *Festuca idahoensis*), resource availability (high or low) and herbivory (elicited or not elicited) to determine the relative influence of these factors on *C. maculosa *growth and production of defense compounds. Methyl jasmonate (MeJA) was used as an elicitor to simulate insect herbivory, as the jasmonate biosynthesis and signaling pathways are known to be induced under conditions of biotic stress [35,36]. Additionally, MeJA can serve as a volatile warning signal of future herbivore attack for plant neighbors, and leads to increased production of defense-related compounds [8,37-40].

To further investigate the findings of our greenhouse experiment in a field setting, we utilized a non-targeted metabolomics analysis to explore chemical differences between naturally occurring *C. maculosa *individuals in two different stand types (conspecific versus heterospecific). Although metabolomics studies have generally been used in highly controlled experiments and applied to genetically uniform model species (but see [41]), we were interested in investigating the utility of this tool using a non-model organism in a field biology setting.

This study not only provides support for the idea that *C. maculosa *individuals modify their physiology and biochemistry based on the identity of their plant neighbor, but it further demonstrates the utility of metabolomics as a tool for field biologists.

## Results

### Greenhouse study

Leaf phenolic content and biomass accumulation in *C. maculosa *individuals were both influenced by various combinations of the three factors tested; plant neighbor identity, nutrient level and whether or not the plants were elicited with MeJA. An overall ANOVA revealed significant interactions between plant neighbor identity and MeJA elicitation for both response variables (phenolics p < 0.0001, biomass, p = 0.0059, Additional file 1 Table S1).

**Figure 1:**
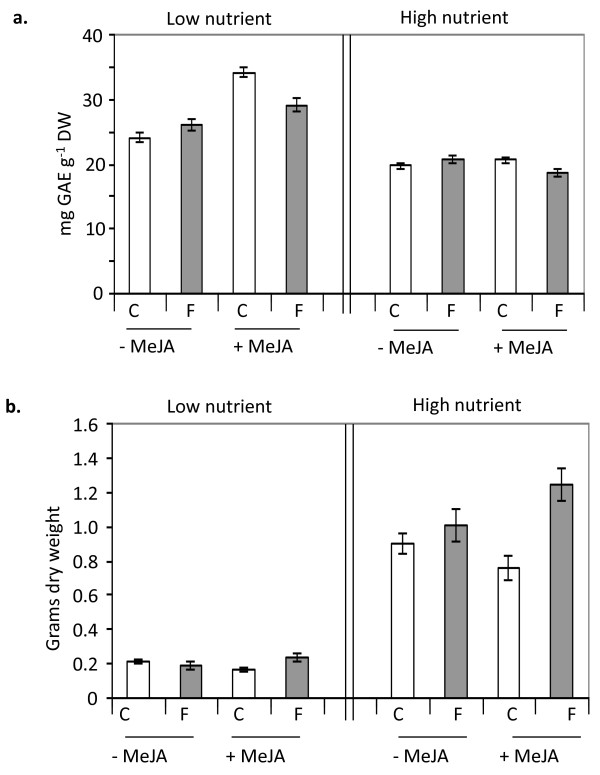
**Total phenolics accumulation and plant biomass in greenhouse experiment**. *Centaurea maculosa *plants were paired with either a conspecific (*C. maculosa*; C) or heterospecific (*F. idahoensis*; F) neighbor and grown in high or low nutrient conditions in the greenhouse. Half of the plant pairs in each nutrient condition were elicited with MeJA (+MeJA) to simulate herbivory. **Panel a: **Accumulation of total phenolics was analyzed in *C. maculosa *leaf tissues using the Folin-Ciocalteu method, values are expressed as mg gallic acid equivalents (GAE) per gram dry weight of plant tissue. **Panel b: **Total dry weight of leaves and roots. Bars represent mean values with standard errors. Refer to pair-wise comparisons (Table 1) for t and p values for significant comparisons.

Due to large differences in both biomass and leaf phenolic content resulting from different levels of resource availability, the data for each nutrient level (low or high) were also analyzed in separate ANOVAs (Table 1). This analysis revealed a significant interaction between plant neighbor identity and MeJA elicitation at both nutrient levels for both response variables (phenolics p < 0.007 and biomass p < 0.026, Table 1). Pair-wise comparisons of this interaction revealed a response that was consistent for both nutrient levels (Table 2). In non-elicited conditions, there were no significant differences between *C. maculosa *leaf phenolic content or in total biomass due to plant neighbor identity (Fig. 1, Table 2; -MeJA, C versus F). However, when the plants were elicited with MeJA, *C. maculosa *individuals accumulated a significantly greater amount of total phenolics and exhibited reduced biomass when growing with a conspecific (*C. maculosa*) versus a heterospecific (*F. idahoensis*) neighbor (Fig. 1, Table 2; +MeJA, C versus F).

**Table 1:** *Centaurea maculosa *total phenolics and total biomass.

Total phenolics			
***Nutrient level***	***Effect***	***F***	***p***

low	Neighbor	3.20	0.0755
	
	Elicitation	58.44	<0.0001
	
	N*E	16.28	<0.0001

high	Neighbor	0.72	0.3964
	
	Elicitation	1.64	0.2029
	
	N*E	7.52	0.0069

**Total biomass**			

***Nutrient level***	***Effect***	***F***	***p***

low	Neighbor	0.99	0.3242

	Elicitation	0.00	0.9917
	
	N*E	6.00	0.0182

high	Neighbor	12.99	0.0008
	
	Elicitation	0.34	0.5656
	
	N*E	5.32	0.0257

ANOVA was performed on measures of *C. maculosa *total phenolics and total biomass (dry weight of roots and shoots). F and p values sorted by nutrient condition.

**Table 2:** *Centaurea maculosa *pairwise comparisons.

Nutrient level	Neighbor Identity	Me JA	Neighbor Identity	Me JA	Total Phenolics		Biomass	
					
					t	p	t	p
Low	Centaurea	-	Centaurea	+	10.31	<0.0001	2.17	0.0350
	
	Festuca	-	Festuca	+	2.19	0.0301	1.48	0.1457
	
	*Centaurea*	-	*Festuca*	-	*1.57*	*0.1180*	*1.02*	*0.3140*
	
	*Centaurea*	+	*Festcua*	+	*4.16*	*<0.0001*	*2.46*	*0.0177*

								

High	Centaurea	-	Centaurea	+	1.28	0.2025	1.50	0.1404
	
	Festuca	-	Festuca	+	2.45	0.0155	1.77	0.0843
	
	*Centaurea*	-	*Festuca*	-	*1.34*	*0.1808*	*0.94*	*0.3517*
	
	*Centaurea*	+	*Festcua*	+	*2.45*	*0.0125*	*4.08*	*0.0002*

Test statistics and p values for pair-wise comparisons of the interaction between plant neighbor identity and elicitation with methyl jasmonate (MeJA) in both high and low nutrient conditions.

Although the presence of a conspecific neighbor was consistently correlated with significant increases in leaf phenolic content and significant reductions in biomass accumulation under MeJA elicited conditions, the extent of these changes was influenced by the level of available nutrients (Fig. 1, Additional file 2 Table S2). For instance, when plants were elicited with MeJA under low nutrient conditions, the relative increase in total phenolic accumulation due to a conspecific versus a heterospecific neighbor was 17% (total increase ~5 mg gallic acid equivalents (GAE) per gram dry weight (Fig. 1a left panel; +MeJA, C versus F)), whereas in high nutrient conditions this increase was 10% (total ~2 mg GAE per gram dry weight (Fig. 1a right panel; +MeJA, C versus F)). Plant biomass exhibited a 28% relative decrease in dry weight (total ~65 mg) under low nutrient conditions versus a 39% decrease (total ~488 mg) under high nutrient conditions due to the presence of a conspecific versus heterospecific neighbor when plants were elicited (Fig. 1b; +MeJA, C versus F).

As expected, higher nutrient levels resulted in larger plants (Fig. 1b; left panel versus right panel), and additionally resulted in plants with lower amounts of total phenolics in leaf tissue (Fig. 1a; left panel versus right panel). It was expected that elicitation with MeJA would result in an increase in total phenolic content of *C. maculosa *leaves. However, total phenolics only increased due to elicitation in low resource conditions (Fig. 1a left panel; -MeJA versus +MeJA).

Root to shoot ratios were calculated for *C. maculosa *individuals as an indicator of response to far-red light given off by neighboring plants (Additional file 3 Table S3). Plant neighbor identity did not have a significant impact on *C. maculosa *root:shoot ratios (Additional file 4 Table S4).

### Metabolomic analysis of field plants

To further investigate the findings of our greenhouse experiment under field conditions, we analyzed naturally occurring *C. maculosa *plants, which are commonly found in both conspecific stands (near monoculture) and heterospecific stands (the invasion front, consisting of diverse plant neighbors).

**Figure 2:**
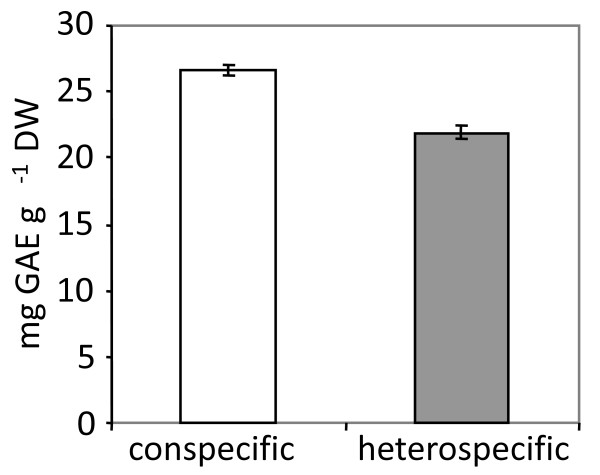
**Total phenolics accumulation of *C. maculosa *plants collected from conspecific and hetetrospecific field stands**. Accumulation of total phenolics was analyzed in freeze dried, field collected *C. maculosa *leaf tissues using the Folin-Ciocalteu method. Values are expressed as mg gallic acid equivalents (GAE) per gram dry weight of plant tissue. Means and standard errors are shown. Stand type significantly affected total phenolics accumulation (t = 6.94, p < 0.0001).

An initial analysis of total phenolics in leaf tissue confirmed increased leaf phenolic content in plants from conspecific versus heterospecific field stands (Fig. 2). We then conducted a non-targeted metabolomics analysis on these *C. maculosa *leaf tissues using Gas Chromotography-Mass Spectrometry (GC-MS) and Ultra-high Pressure Liquid Chromatography - Mass Spectrometry (UPLC-MS). The metabolomics-based analyses of field collected *C. maculosa *leaves from conspecific stands revealed a significantly lower accumulation of many small primary metabolites involved in glycolysis, the tricarboxylic acid cycle, lipid metabolism, and amino acid metabolism in comparison to *C. maculosa *plants growing in more diverse heterospecific stands (Table 3, Additional file 5 Table S5). GC-MS analysis revealed significantly reduced amounts of maleic acid, fumaric acid, succinic acid, fructose and six protein amino acids in conspecific versus heterospecific stands (Table 3). In addition, many metabolites potentially involved in membrane metabolism were decreased in conspecific stands, including phosphoric acid, ethanol amine, glycerol, glycerophosphate and linoleic acid (Table 3). Wax components such as hexacosanol, octacosanol, and hexacosanoic acid were also found at diminished levels in conspecific versus heterospecific stands (Table 3).

**Table 3:** Compounds identified by GC-MS demonstrating significant ANOVA effects from stand type (conspecific or heterospecific).

Metabolites higher in heterospecific stands		
***Compound***	***Fold change (Conspecific/Heterospecific)***	***ANOVA p-value (stand type)***

Glycine	0.702	0.0023

Cytosine	0.370	0.0019

L-Alanine	0.483	<0.0001

L-Aspartic acid	0.562	0.001

L-Threonine	0.578	0.001

L-Proline	0.367	0.0035

Ethanol amine	0.812	<0.0001

Pyroglutamic acid	0.621	<0.0001

4-aminobutyric acid	0.599	0.0029

3-hydroxybenzoate	0.923	0.0077

Glycerol	0.856	0.0096

Catechol	0.818	0.0096

Ribose	0.763	0.0092

Fructose	0.716	0.0013

Fructose	0.733	0.0015

Maleic acid	0.575	0.0005

Succinic Acid	0.781	0.001

Fumaric Acid	0.651	<0.0001

Phosphoric acid (polar)	0.789	0.0064

Phosphoric acid (non-polar)	0.807	0.0094

Glycerophosphate	0.706	0.0032

Phytol	0.743	0.0053

Linoleic acid	0.794	0.0119

Hexacosanol	0.805	0.0057

Hexacosanoic acid	0.738	0.0035

Octacosanol	0.829	0.0064

**Metabolites higher in conspecific stands**		

***Compound***	***Fold change (Conspecific/Heterospecific)***	***ANOVA p-value***

Quinic Acid	1.199	0.0012

Inositol-like	1.586	0.0025

Inositol-like	1.316	0.0001

Galactose	1.245	0.0009

Galactonic acid	1.218	0.0002

Chlorogenic acid	2.080	0.0111

In contrast, multiple secondary metabolites including inositol-like compounds, cholorogenic acid, and quinic acid demonstrated significantly increased accumulation in conspecific stands (Table 3, Additional file 5 Table S5). In addition, several unidentified polar metabolites that eluted from the chromatographic column at longer retention times exhibited increased abundance in plants from conspecific stands (data not shown). Galactose was the only primary metabolite identified that was increased in conspecific versus heterospecific stands (Table 3).

**Figure 3:**
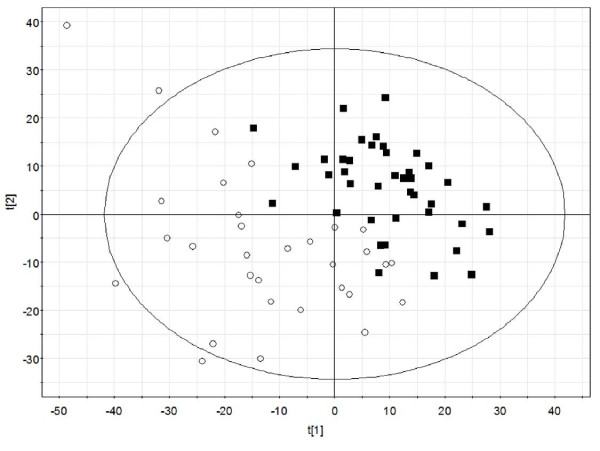
**UPLC-MS analysis allows distinction of samples from conspecific and heterospecific stands**. Samples were processed and analyzed as described in Materials and Methods. The resulting dataset was analyzed by principle component analysis followed by projection to latent structures - discriminant analysis (PLS-DA). The resulting plot demonstrates that individuals from conspecific and heterospecific stands can be distinguished. Open circles indicate samples from heterospecifc stands and filled squares indicate samples from conspecific stands. The x- and y-axes (t[1] and t[2]) represent the first and second PLS components, respectively. Ellipse represents the 95% confidence interval. Randomization of the relative density variable (conspecific versus heterospecifc stands) eliminated predictive power of the PLS-DA model.

Univariate ANOVA of UPLC-MS samples revealed approximately 100 mass spectral signals that were significantly affected by stand type (conspecific versus heterospecific) at *p *< 0.01 (Additional file 5 Table S5). Although many of the features were unable to be identified, the sesquiterpene lactone cnicin, an herbivore defense compound primarily found in *Centaurea *species, accumulated to significantly higher levels in plants from conspecific stands based on comparison to an authentic cnicin standard (Phytoplan, Heidelberg, Germany) (Additional file 5 Table S5, [M+H]+ m/z 379.18 at 10.78 minutes, p = 0.0048). Multivariate analyses of the UPLC-MS data demonstrate that samples from conspecific and heterospecific stands can be distinguished (Fig. 3).

### Herbivory rates and other field parameters

The metabolomics results indicated that plant neighbor identity might affect individual plant metabolism in the field, as metabolites accumulated to significantly different levels and multivariate data analysis was able to separate samples based on stand type (Fig. 3). However, factors such as resource availability and herbivory also impact the extent to which *C. maculosa *plants allocate resources towards primary versus secondary metabolism, as was demonstrated in the greenhouse experiment. Thus, it was important to investigate if other factors in the field that might correlate with the relative density of *C. maculosa *could have contributed to or confounded the metabolomics results.

To determine if resource availability was correlated with stand type under field conditions, three soil samples were taken from the base of three randomly selected *C. maculosa *plants per stand type at each site. Analyses of soil characteristics revealed no significant differences between the conspecific and heterospecific *C. maculosa *field stands (Additional file 6 Table S6). In general, these parameters differed more due to site than to stand type (Additional file 6 Table S6).

To investigate the potential influence of herbivore damage on the *C. maculosa *metabolomics results, evidence of leaf herbivory was noted for all plants sampled. A portion of individual plants sampled from both stand types experienced some form of leaf herbivory. However, leaf herbivory data collected from conspecific and heterospecific field stands at the time of sampling did not improve ANOVA model fit when included as a covariate, suggesting that herbivory was not a major factor influencing the results.

Although leaf herbivory damage data taken at the time of sampling did not help explain the metabolomics results, it was possible that the presence of unseen herbivores caused changes in the *C. maculosa *leaf metabolome, as a variety of specialist herbivores have been introduced into North America that feed on *C. maculosa *roots or flowers as opposed to leaf tissue.

**Figure 4:**
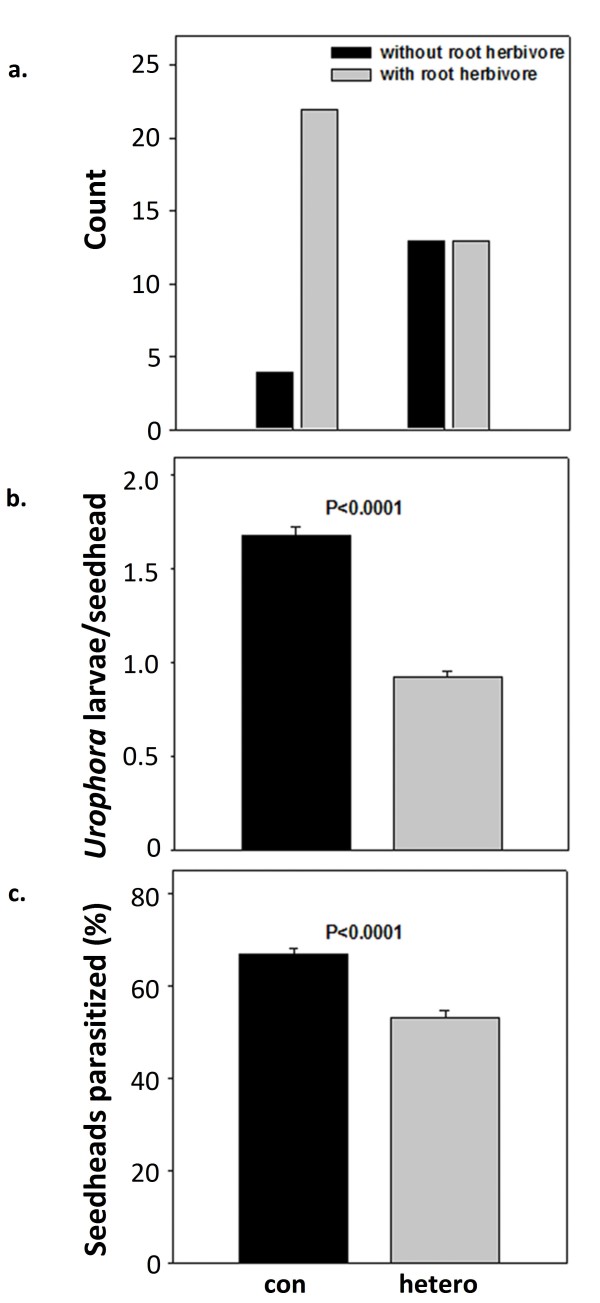
**Plants from conspecific stands undergo increased specialist herbivory compared to plants from heterospecific stands**. **Panel a: **Numbers of plants with and without the root herbivore, *Agapeta zoegana*, or evidence of damage from root herbivores in conspecific (high density) and heterospecific (low density) stands of *C. maculosa*. For each density level n = 26 plants. Chi-square = 7.08, *p *= 0.008. **Panel b: ***Urophora spp*. found in *C. maculosa *stands. **Panel c: **Seed heads parasitized by *Urophora spp*. in *C. maculosa *stands.

We conducted an independent analysis of specialist herbivore abundance and damage in *C. maculosa *stands. Specialist root and flower herbivores occurred more frequently in conspecific versus heterospecific stands. Root damage by the specialist herbivore *Agapeta zoegana *was higher in conspecific than in heterospecific stands of *C. maculosa *(Fig. 4a). Additionally, the abundance of the flower head herbivore *Urophora spp*. was higher in conspecific stands of *C. maculosa *(Fig. 4b) and a higher percentage of seed heads were parasitized by *Urophora *in conspecific stands (Fig. 4c).

## Discussion

The results of the field metabolomics study tend to reflect those found in the greenhouse experiment. Field collected *C. maculosa *plants with heterospecific neighbors allocate more resources towards the production of primary metabolites which are crucial for plant growth, and allocate fewer resources to secondary metabolite production. Similarly, in the greenhouse elicited *C. maculosa *plants with heterospecific (*F. idahoensis*) neighbors allocated more resources towards biomass production, and fewer resources towards production of leaf phenolic compounds. Field collected *C. maculosa *individuals with conspecific neighbors allocate more resources towards production of secondary metabolites. Likewise, in greenhouse conditions, elicited *C. maculosa *plants with conspecific neighbors allocated more resources towards production of phenolic compounds and fewer towards biomass production.

Secondary metabolites, particularly phenolic compounds are often implicated in plant anti-herbivore defense mechanisms [42-47], and have been considered important factors in multiple hypotheses of plant defense against herbivores [48-50]. Thus, the increased levels of secondary metabolites in *C. maculosa *grown with conspecific neighbors may reflect an increase in herbivore defense response. Galactose was the only primary metabolite identified that was increased in conspecific versus heterospecific stands (Table 3). Interestingly, galactose is reported to have defensive properties in spruce trees against the herbivore western spruce budworm when compared to the other sugars fructose and glucose [51].

In the greenhouse experiment, differences in resource allocation due to plant neighbor were only identified under conditions of simulated herbivory, and there was a strong interaction between these two factors. However, under field conditions, leaf herbivory data taken on site did not improve ANOVA model fit, initially suggesting that plant neighbor identity may play a larger role than herbivory in *C. maculosa *metabolism in the field. In a separate study, greater numbers of *C. maculosa *conspecifics were positively correlated with root and flower specialist herbivore damage and abundance. There is some evidence to suggest that below ground herbivory can increase production of secondary compounds present in plant leaves [52,53], and that plant defenses can be up-regulated in tissues distant from the site of herbivore attack [52]. Thus, we cannot rule out the possibility that an increased rate of specialist herbivory in conspecific stands led to the observed increases in leaf secondary metabolites.

Although measures of specialist herbivory confounded the potential link between plant neighbor identity and defensive chemistry in the field study, the results of the greenhouse experiment suggested that plant neighbor identity was an important factor in *C. maculosa *defense response. However, a variety of resource-related factors, not typically linked to specific neighbor identity, are known to influence plant metabolism and should mentioned in regards to plant neighbor-induced changes in our experiment.

For instance, differences in light amount or quality in the environment are involved in both plant competition and defense response. Plants are able to sense the presence of plant competitors by detecting changes in light quality (particularly red: far-red) through phytochrome pathways [54]. Increases in far-red (FR) radiation impact plant secondary metabolite accumulation [55], and can lead to an attenuated defense response resulting in increased levels of herbivory, fewer phenolic compounds and reduced expression of genes in the jamonate pathway [14,56]. In the current greenhouse study, *C. maculosa *plants appeared to be experiencing similar amounts of FR radiation between neighbor treatments, as root to shoot ratios [a classic indicator of FR induced morphological changes [12]] did not change significantly due to neighbor identity within nutrient treatments (Additional file 3 Table S3, Additional file 4 Table S4). Thus, although changes in light quality are able to impact plant metabolism and defense response, differences in FR-response do not explain observed differences in biomass or phenolics in the greenhouse.

In some plants, ultraviolet (UV)-B radiation triggers JA accumulation [57], induces signaling pathways similar to those involved in plant response to herbivory [41,58], and can lead to increases in plant phenolic compounds [41]. The majority of UV-B radiation is blocked inside greenhouses, and therefore UV-B is not presumed to be a factor in the results of the greenhouse experiment. Site geographical features are likely the most important factors determining plant exposure to UV-B in the field, but it is possible that stand type (plant neighbor identity) could influence UV-B amounts (through shading, etc).

Nutrient availability can directly influence plant metabolism and is integrated into multiple plant-defense hypotheses [48,50]. In the greenhouse study, nutrient condition directly impacted mean levels of both biomass and leaf phenolic contents. However, the response trends identified for the interaction between plant neighbor identity and elicitation were the same in both the high and low nutrient condition. Soil nutrient availability was highly variable in the field experiment (Additional file 6 Table S6), yet the results paralleled the general trends of the greenhouse experiment in that metabolic profiles differed due to stand type.

Changes in resource ability (nutrients and light) can have profound effects on plant metabolism and defense response, but did not appear to be key factors influencing the interaction between plant neighbor identity and elicitation identified in the current study. This suggests that *C. maculosa *individuals utilized some other form of perception to differentiate between plant neighbors and subsequently modify their defense response.

A variety of other potential plant neighbor recognition mechanisms have been posited in the literature, including the perception of plant volatile organic compounds (VOCs) [37,38,40,59,60], root exudates [61], oscillatory signals [7], and physical contact between roots or leaves [62]. Of these possible mechanisms of plant to plant signaling, VOC-mediated communication (often referred to as 'eavesdropping') has received the most attention, as multiple recent studies have demonstrated direct and indirect VOC-induced plant defense response (see [63] for a brief review). MeJA, the elicitor used in our greenhouse experiment, is a naturally occurring VOC that, in sagebrush, can provoke defense response in conspecific plant neighbors, and to varying degrees in heterospecific plant neighbors [8,37-40,64]. However, the extent of VOC specificity and the fitness consequences of VOC-mediated communication for 'emitter' and 'receiver' plants remain under debate [63,64]. It is not clear if *C. maculosa *utilizes VOCs in plant communication; however, it is interesting to speculate that *C. maculosa *individuals modify their VOC bouquet in relation to plant neighbor identity leading to neighbor specific changes in defensive chemistry.

Other evidence suggests that *C. maculosa *exhibits specificity in its response to plant neighbors [26]. Expression of some *C. maculosa *genes are up or down regulated due to the presence versus absence of a plant neighbor; while other genes appear to be regulated in a neighbor specific fashion [26]. Although the experiment of Broz *et al*. (2008) did not evaluate gene expression in *C. maculosa *paired with conspecifics or in situations of herbivory, it lends support to the idea that *C. maculosa *is able to differentially sense and respond to specific plant neighbors. In *Solanum nigrum*, Schmidt and Baldwin (2006) found that the presence of heterospecific competitors (versus no competitors) results in decreased expression of primary metabolism genes, both with and without elicitation by MeJA [65]. In addition, MeJA elicited a response consistent with a trade-off between growth and defense (reduced expression of primary metabolism genes; increased expression of defense-related genes), regardless of the competitive situation [65]. In the greenhouse study presented here, MeJA elicitation led to this type of response only when plants were paired with conspecific neighbors. Although gene expression is not always a good indicator of metabolite concentrations, which may help explain this discrepancy, our results suggest that plant neighbor identity can have important impacts on the presumed trade-off between plant growth and defense. Clearly, further investigations are needed to identify the recognition mechanism and response system involved in *C. maculosa *plant-neighbor communication.

Although more evidence is needed to provide a direct link between plant neighbor identity and defense response under field conditions, it is plausible that a modification of a defense strategy based on plant neighbor identity would confer evolutionary advantages. For instance, high conspecific plant density often correlates with higher attack rates by consumers [23], as was identified for *C. maculosa *in our field analysis of specialist herbivores. Individual plants growing in conspecific stands are more likely to be subject to herbivore attack than those growing in diverse plant communities. Thus, in a conspecific stand, a defensive strategy involving the accumulation of chemical herbivore deterrents is likely to be more effective than a strategy based on growth. In a heterospecific stand where the probability or occurrence of herbivore attack is lower, factors such as plant competition for light or nutrients could have larger impacts on individual success. Thus, investing resources in growth rather than defense may be more effective over time in a diverse plant community, even when herbivores are present.

## Conclusions

Although the perception of and response to neighbors is widely recognized in other taxa ranging from microorganisms to mammals [1-6], it remains understudied in the field of plant biology. Our results indicate that greenhouse-grown *C. maculosa *individuals modify their defensive chemistry based on the identity of their plant neighbor. In addition, *C. maculosa *individuals were found to exhibit different metabolic profiles in the field based on stand type (heterospecific or conspecific), which is likely due to a combination of factors including plant neighbor identity and rates of specialist herbivory. Whether or not a majority of plant species are able to differentially sense and respond to different plant neighbors remains to be determined. If plants are indeed capable of these processes it will have large implications for both the study and human management of ecological systems.

## Methods

### Greenhouse experiment

#### Greenhouse experimental design and sampling

*Centaurea maculosa *seeds were collected from field populations near Missoula, MT, USA and *Festuca idahoensis *Elmer seeds were obtained from Wind River Seed Company (Manderson WY, a distributor of native seeds from the United States). *F. idahoensis *is a native North American grass species commonly found in heterospecific stands of *C. maculosa*. In late April 2008, cone-tainer pots (volume 164 cm^3 ^each) were filled with a mix of 2 parts sand (Play Sand obtained from US Mix, Denver CO) and one part soil clay conditioner (100% arcillite obtained from Schultz, Bridgeton MO). Pots were placed into racks in the greenhouse, flooded with water to settle soil media, seeded and covered with plastic wrap to maintain a humid environment. The factors in the experimental design were plant-neighbor identity (3; *C. maculosa-C. maculosa, C. maculosa-F. idahoensis*, or *F. idahoensis-F. idahoensis*) nutrient level (2; high or low) and elicitation (2; elicited with MeJA or not elicited), and for each combination there were 8 repetitions giving a total of 96 experimental units. Three seeds of each competitor were added to the appropriate pots. Once seedlings were established (~2 weeks) the plants were thinned to two total plants per pot. On May 12, 2008 the plants were randomized in a complete block design, with nutrient level as the blocking factor. In the high nutrient condition plants were watered with 1/2 strength Hoaglands solution twice per week and in the low nutrient condition plants were watered with 1/20 strength Hoaglands solution twice per week. Approximately 20 mL of nutrient solution was put into each pot on Mondays and Fridays, and plants were watered on Wednesdays over the course of the experiment. On July 18^th^, the pots were un-randomized and re-randomized based on two blocking factors: nutrient level and elicitation. On this day, (two weeks before harvest) half the pots were elicited by spraying both plants in the pot with a 0.5 mM methyl jasmonate (MeJA) solution until they were thoroughly soaked. The MeJA solution was made by adding 57.3 μL of methyl jasmonate 95% (Sigma #392707, mw 224.3, Saint Louis, MO) to 25 mL of methanol to create a 10 mM solution. Fifteen mL of the 10 mM solution was brought up to 300 mL with water to make a 0.5 mM solution. For a control, 15 mL of MeOH were added to 285 mL of water. Plants were treated with 0.5 mM MeJA solution again one week before harvest and one day before harvest. The control plants were sprayed with a water-methanol solution. During MeJA treatments, racks (blocks) of pots were separated by at least three feet and were further separated by a temporary barrier to ensure that control plants did not come into contact with the MeJA solution.

Plants were removed from pots and roots were placed in water to rinse away soil media and separate the two plants. Total root samples from each plant were placed into individual envelopes and dried at 60°C for two days. Shoot samples from each plant were placed in individual tubes and placed at -80°C for at least four hours, after which they were freeze dried under a vacuum at -75°C for two days. All dry shoot and root samples were weighed to the nearest mg. Shoot samples were ground with a coffee grinder to a fine powder and stored at 4°C.

#### Total phenolics assay

For each plant sample, one mL of 80% acetone was added to 20 mg of ground plant tissue, vortexed 30 seconds, rotated at 250 rpm in the dark for 15 min, vortexed briefly, and centrifuged at 10,000 rpm at 4°C for 15 min. A 100 μL aliquot of supernatant was removed and stored at -20°C overnight. The aliquot was brought to a total volume of 1 mL with distilled water, vortexed, and used in a colorimetric microplate assay for phenolics determination.

The Folin-Ciocalteu assay was used to determine the amount of total phenolics in all samples, using gallic acid to create a standard curve. Briefly, 35 μL of each sample was mixed with 150 μL of 0.2 M Folin-Ciocalteu reagent and incubated at room temperature for 5 min, after which 115 μL of saturated (7.5% w/v) sodium bicarbonate was added to the reaction. The reaction was mixed briefly, incubated at 45°C for 30 min, and then allowed to cool to room temperature for 60 min. Absorbance at wavelength 765 was read in a SPECTRA max plus 384 microplate reader (Molecular devices, Sunnyvale CA). All samples were run in triplicate. Total phenolics were calculated as gallic acid equivalents (GAE) per gram of sample dry weight.

#### Statistical analyses of greenhouse experiment data

Analyses of total biomass and phenolics data were performed using the mixed procedure in the SAS 9.1 program. Total biomass data were not normally distributed, and were log transformed to normalize distribution. However, significance of interactions and pair-wise comparisons did not change with log transformation of the data. Phenolics data were normally distributed. Root to shoot ratios were log transformed to normalize distribution. ANOVAs were computed for the entire data set, and separately by nutrient condition. Pair-wise comparisons between means were made to determine significant differences between conditions of interest using Fischers LSD.

### Field Experiment and Metabolomic Analysis

#### Field sites

For metabolomic analyses, two field sites were chosen near Missoula, Montana and plants were collected in late May 2006 (Site A, Beavertail site: 12T 0301244E 5177747N and Site B: 12T 273192E 5193062N). At both sites the density of *C. maculosa *varies from low to very high densities. Herbivore response to *C. maculosa *density was measured at the Mt. Sentinel site and at a third site, the North Hills of Missoula, Montana. Plants sampled were categorized as growing under low density conditions if individual plants occurred at < 0.1 per m^2^. Plants were categorized as high density if they occurred at > 15 per m^2^.

In general, a wide variety of native (and some invasive) plant species typically co-occur with *C. maculosa *in heterospecific sites near Missoula, Montana (Giles Thelen, University of Montana, personal communication). Although vegetation surveys noting the exact species types and amounts were not performed at sites sampled during the field experiment, similar sites in Montana tend to display a large amount of variability in regards to plant functional group both within and between heterospecific sites [66]. A variety of native grasses, forbs and legumes were found to co-occur with *C. maculosa *in the heterospecific sites sampled in the current study (Corey Broeckling, personal observation).

#### Field experimental design and sampling

Sampling was conducted using a block design, with day and site serving as blocks. Sampling was conducted on two consecutive days. Each of two sites was visited in opposing order each day between 11:00 and 15:00 to minimize diurnal effects on metabolite accumulation data. On each sampling day, eight plants from conspecific stands and eight plants from heterospecific stands were sampled at each site. Plants from high density, conspecific stands were those that contained at least ten conspecifics within a 1.0 m radius of the sample plant. Plants from low density, heterospecific stands were those that contained fewer than three conspecifics within a 1.0 m radius. This design resulted in a sampling regime composed of two sites visited on two consecutive days, for a total of 32 samples each of plants from conspecific and heterospecific stands, and a total of 64 samples. For each sample, we also collected data for factors that might affect metabolism including herbivory, distance to the nearest conspecific, distance to the nearest other species, and number of conspecifics within a 30 cm radius. Hourly temperature and relative humidity was obtained from the NOAA website for Missoula, MT. All of these variables were added to the basic ANOVA model as covariates, but failed to improve model fit and were thus not included in model used for the final analysis.

Individual *C. maculosa *rosettes without current-year flowering stalks were selected for metabolite analyses. However, for the sake of consistency, only plants with dried flowering stalks from the previous year were sampled, ensuring that samples were taken from individuals in at least their second year of growth. Non-senescent, fully expanded mature leaves were harvested from the rosette and immediately frozen on dry ice, after which they were transferred to an -80°C freezer. Individual samples were processed by grinding them to a fine powder in liquid nitrogen and freeze drying.

Soil samples were collected approximately 10 cm from the base of three randomly selected high density and low density plants at each site. Soil was sampled to a depth of approximately 10 cm, and was immediately frozen on dry ice. Soil nutrient analysis was performed by the Soil, Water and Plant testing laboratory at Colorado State University using standard methods.

#### Total Phenolics Assay

Analysis of total phenolics in field sampled leaf tissues was performed as described above (Methods; Greenhouse Experiment, Total Phenolics Assay). Analysis of the data was performed using the t-test function in Microsoft Excel (n = 32 plant samples for each stand type; t = 6.94, p < 0.0001).

#### Metabolome analysis

Metabolome analysis for GC-MS was conducted essentially as previously described [67]. For GC-MS plant metabolome analysis, 6.0 mg of dried tissue was extracted with 1.5 mL CHCl_3 _(with internal standard - IS) for 60 min at 37°C. After 60 min, 1.5 mL of water (with IS) was added to form a biphasic solvent system. This mixture was thoroughly vortexed and incubated for an additional 60 min at 37°C. The samples were then centrifuged at 3000 × g for 30 min to separate the solvents. One mL of the CHCl_3 _fraction was collected and transferred to an autosampler vial - this comprises the non-polar fraction. CHCl_3 _was evaporated under a gentle flow of nitrogen gas. The dried sample was derivatized in 70 μL of pyridine and 30 μL MSTFA at 50°C for one hour to generate trimethylsilyl derivatives. One mL of the aqueous fraction was collected and transferred to an autosampler vial - this comprises the polar fraction.

The aqueous extract was held at -80°C until it was dried in a vacuum centrifuge at ambient temperature. The dried aqueous extract was derivatized with 120 μL of pyridine with 15 mg/mL methoxyamine HCl for 1 hr at 50°C, with occasional vortexing and sonicating in a water bath. One hundred twenty μL of MSTFA was then added and incubated at 50°C for 30 min to trimethylsilylate the polar compounds.

Separation was performed on a 60 m DB5-MS (J&W Scientific, 0.25 mm ID, 0.25 μm film thickness) column. Separation was achieved with a temperature program of 80°C for two min, then ramped at 5°C min^-1 ^to 315°C and held for 12 min and a constant flow of 1.0 ml min^-1^. Mass data was collected on an Agilent 5973 single quadrupole mass spectrometer using electron impact ionization. One μL of derivatized non-polar fraction was injected onto an Agilent 6890 GC using a 1:1 split ratio. A 1.0 μL portion of the aqueous fraction was analyzed in the same manner, except a 15:1 split ratio was used. All identifications were made by comparison to a custom authentic standard library by comparison of retention time and mass spectral data.

For UPLC-MS analysis, 40 mg of freeze dried and homogenized leaf tissue was extracted twice in 70% methanol in water containing 0.1 μg/μL 4-methylumbelliferone (internal standard). The extracts were centrifuged to remove particulate material and pooled. Samples were held at 10°C during the analysis. One microliter injections were separated by reverse phase chromatography using an Acquity UPLC™ (Waters Corporation, Milford, MA, USA). Solvent and column parameters are as follows: Solvent A = 95:5 H2O:methanol (Fisher Optima LC/MS grade) + 0.1% formic acid (Fluka, LC/MS grade); Solvent B = 100% methanol + 0.1% formic acid; column = 1.0 × 100 mm Waters Acquity UPLC™ BEH C18 1.71 μm particle size; column temperature = 40°C. The solvent gradient parameters were as follows: Flow rate: 0.140 mL/min; 0 to 2 min: Solvent A 100%; 2 to 22 min: Solvent A 100% to Solvent B 100%; 22 to 25 min: Solvent B 100%; 25 to 28 min: Solvent B 100% to Solvent A 100%; 28 to 30 min: Solvent A 100%. A short gradient was run between each sample to ensure no carry-over and equilibrate the column. The equilibration gradient characteristics were as follows: Flow rate: 0.140 mL/min; 0 to 0.1 min: Solvent A 100%; 0.1 to 5 min: Solvent A 100% to Solvent B 100%; 5 to 8 min: Solvent B 100%; 8 to 11 min: Solvent B 100% to Solvent A 100%; 11 to 20 min: Solvent A 100%.

Effluent from the UPLC system was infused directly into a Waters Micromass Micro quadrupole orthogonal acceleration time-of-flight mass spectrometer (Q-TOFMS) via electrospray ionization (ESI), in the positive ion mode using the following operating parameters: Capillary: 3000 V; Sample cone: 35 V; Extraction cone: 2 V; Collision cell: 7 eV, 20 psi pressure (argon); Source temperature: 130°C; Desolvation temperature: 300°C; Desolvation gas flow: 400 L/h. Sodium formate was used to calibrate the Q-TOF across the mass range of detection. Leucine enkaphalin was introduced via a secondary LockSpray™ positive ion ESI source as a mass standard to improve the accuracy of collected mass values. Mass data were collected in real-time centroid mode, creating centered measurements for each scan. The measured mass resolution was measured to be 5000 (FWHM). Both the UPLC and Q-TOF were controlled by Waters MassLynx software (v4.1). Identification of cnicin was based on comparison of retention time and mass spectrum to authentic standard (Phytoplan cat# 2113.98, Heidelberg, Germany).

#### Metabolomic data extraction and statistical analysis

GC-MS data was processed using AMDIS [68] for peak detection from multiple randomly selected samples and quantitative peak area data extracted using default settings in MET-IDEA [69]. Redundant peaks were removed and data were normalized to internal standard peak area to adjust for sensitivity drift of the instrumentation. CDF plots were generated and ANOVA was conducted in JMP v.5.1 (SAS Institute, Cary, NC, USA).

UPLC-MS data were extracted and aligned with Waters MarkerLynx software (v4.1) using the following parameters: retention time range: 0 - 24 min; mass range: 50 - 1000 Da; "apex Track peak parameters": automatically calculated peak width and baseline noise with no smoothing; "collection parameters": intensity threshold: 20 counts; mass window: 0.07 Da; retention time window: 0.1 min. Analyte features were labeled by their retention time and mass, and exported to Umetrics SIMCA-P v11 (Umetrics, Umeå, Sweeden) for multivariate analysis. Pareto scaling was applied to all data. Principal components analysis (PCA) was used as an unsupervised method for observing sample grouping. Partial least-squares projection to latent structures-discriminant analysis (PLS-DA) was used to classify and group related samples.

### Specialist herbivore field analysis

Signs of herbivory were measured in *C. maculosa *plants from heterospecific stands (< 0.1 plant/m^2^) and conspecific stands (> 15 plants/m^2^) at two additional sites. We collected and dissected five seedheads per plant, and counted the number of specialist *Urophora *seedhead gallflies (Insecta: Diptera: Tephritidae) larvae, pupae and empty pupal cases. We also measured the proportion of *C. maculosa *plants damage from the specialst root herbivores *Cyphocleonus achaetes *(Fahraeus) (Insecta: Coleoptera: Curculionidae) and *Agapeta zoegana *(Linnaeus) (Insecta: Lepidoptera: Cochylidae). A total of 52 plants were excavated and the taproot dissected to look for insects or evidence of recent insect damage. The numbers of plants with insects and evidence for damage were pooled for each stand type and the proportions with and without evidence of root herbivory were compared in regards to stand type with Chi-square analysis.

## Authors' contributions

AKB: Conceived, designed, performed and analyzed data from greenhouse experiment; wrote manuscript. CDB: Conceived, designed, performed and analyzed data from field metabolomics experiment; wrote manuscript. CD: Performed GC metabolomics experiment. MRL: Performed UPLC-MS metabolomics experiment. EG and RMC: Designed and performed field herbivory experiment. LWS: Performed GC metabolomics experiment. JMV: Conceived greenhouse and field metabolomics experiments. All authors edited and approved this manuscript.

## Supplementary Material

Additional file 1 **Table S1. *Centaurea maculosa *greenhouse experiment overall ANOVA**. Total phenolics and total biomass ANOVA F and p values are given for all main effects and interactions. Click here for file

Additional file 2 **Table S2. Greenhouse experiment means and standard errors**. Means and standard errors for total biomass (mg dry weight) and total phenolics (mg gallic acid equivalents (GAE) per gram dry tissue) of *C. maculosa *grown in one of two nutrient conditions (low or high), with one of two neighbor plants (Centaurea or Festuca), and either elicited (+) or not elicited (-) with Methyl Jasmonate (MeJA). For each reported mean, n = 8. Click here for file

Additional file 3 **Table S3. Root to shoot ratios and standard errors of *C. maculosa***. Root to shoot ratios and standard errors for C. maculosa plants grown in the greenhouse experiment. Click here for file

Additional file 4 **Table S4. *Centaurea maculosa *greenhouse experiment overall ANOVA for root to shoot ratios**. Root shoot ratios were log transformed to fit normality assumptions. ANOVA F and p values are given for all main effects and interactions. Click here for file

Additional file 5 **Table S5. Mean, standard error, and ANOVA results for all features identified by UPLC-MS demonstrating significant ANOVA effects from relative density**. Mean, standard error, and ANOVA results for all features identified by UPLC-MS demonstrating significant ANOVA effects from relative density. (H, high density, conspecific stands; L, low density, heterospecific stands). Click here for file

Additional file 6 **Table S6. Soil physiochemical properties**. Soil properties in heterospecific (L, low density of *C. maculosa*) and conspecific (H, high density of *C. maculosa*) stands. Standard deviations are noted in parenthesis for each measurement. None of the measurements were significantly different between stand type at p = 0.05. Click here for file
