# Supplementary material for: Plant neighbor identity influences plant biochemistry and physiology related to defense
Source: BMC Plant Biol. 2010 Jun 17;10:115. doi: 10.1186/1471-2229-10-115 (PMC3095278; doi:10.1186/1471-2229-10-115)
Supplement: Additional file 1 — Table S1. Centaurea maculosa greenhouse experiment overall ANOVA. Total phenolics and total biomass ANOVA F and p values are given for all main effects and interactions. [file 1471-2229-10-115-S1.DOC]

**Additional File 1 - Table S1. *Centaurea maculosa* greenhouse experiment overall ANOVA.**

Total phenolics and total biomass ANOVA F and p values are given for all main effects and interactions.

| **Total phenolics** | | |
| --- | --- | --- |
| *Effect* | *F* | *p* |
| Nutrient level | 269.39 | <0.0001 |
| Neighbor | 3.84 | 0.0509 |
| Elicitation | 32.96 | <0.0001 |
| Nutrient level*Neighbor | 1.10 | 0.2951 |
| Nutrient level*Elicitation | 50.58 | <0.0001 |
| Neighbor*Elicitation | 23.63 | <0.0001 |
| Nutrient level*Neighbor*Elicitation | 3.69 | 0.0556 |
| **Total biomass** | | |
| *Effect* | *F* | *p* |
| Nutrient level | 345.23 | <0.0001 |
| Neighbor | 14.20 | 0.0003 |
| Elicitation | 0.32 | 0.5724 |
| Nutrient level*Neighbor | 11.01 | 0.0013 |
| Nutrient level*Elicitation | 0.33 | 0.5692 |
| Neighbor*Elicitation | 7.96 | 0.0059 |
| Nutrient level*Neighbor*Elicitation | 2.94 | 0.0899 |
