# Supplementary material for: Plant neighbor identity influences plant biochemistry and physiology related to defense
Source: BMC Plant Biol. 2010 Jun 17;10:115. doi: 10.1186/1471-2229-10-115 (PMC3095278; doi:10.1186/1471-2229-10-115)
Supplement: Additional file 2 — Table S2. Greenhouse experiment means and standard errors. Means and standard errors for total biomass (mg dry weight) and total phenolics (mg gallic acid equivalents (GAE) per gram dry tissue) of C. maculosa grown in one of two nutrient conditions (low or high), with one of two neighbor plants (Centaurea or Festuca), and either elicited (+) or not elicited (-) with Methyl Jasmonate (MeJA). For each reported mean, n = 8. [file 1471-2229-10-115-S2.DOC]

**Additional File 2 - Table S2. Greenhouse experiment means and standard errors.**

Means and standard for total phenolics (mg gallic acid equivalents (GAE) per gram dry tissue) and total biomass (mg dry weight) of *C. maculosa* grown in one of two nutrient conditions (low or high), with one of two neighbor plants (Centaurea or Festuca), and either elicited (+) or not elicited (-) with methyl jasmonate (MeJA). For each reported mean, n=8.

| **Nutrient level** | **Competitor Identity** | **MeJA** | **Total Phenolics**  **(mg GAE g^-1^ dry weight)** | | **Biomass**  **(mg dry weight)** | |
| --- | --- | --- | --- | --- | --- | --- |
|  |  |  | **mean** | **se** | **mean** | **se** |
| Low | Centaurea | - | 24.12 | 0.71 | 214.30 | 15.66 |
|  | Festuca | - | 26.05 | 1.00 | 186.70 | 22.14 |
|  | Centaurea | + | 34.18 | 0.67 | 167.60 | 14.76 |
|  | Festuca | + | 29.16 | 1.00 | 233.00 | 22.14 |
|  |  |  |  |  |  |  |
| High | Centaurea | - | 19.80 | 0.47 | 903.60 | 61.96 |
|  | Festuca | - | 20.84 | 0.64 | 1,010.70 | 95.49 |
|  | Centaurea | - | 20.61 | 0.45 | 760.80 | 72.19 |
|  | Festuca | + | 18.63 | 0.64 | 1,249.10 | 95.49 |
