# Supplementary material for: Plant neighbor identity influences plant biochemistry and physiology related to defense
Source: BMC Plant Biol. 2010 Jun 17;10:115. doi: 10.1186/1471-2229-10-115 (PMC3095278; doi:10.1186/1471-2229-10-115)
Supplement: Additional file 3 — Table S3. Root to shoot ratios and standard errors of C. maculosa. Root to shoot ratios and standard errors for C. maculosa plants grown in the greenhouse experiment. [file 1471-2229-10-115-S3.DOC]

**Additional File 3 - Table S3. Root to shoot ratios and standard errors of *C. maculosa.***

Root to shoot ratios and standard errors for C. maculosa plants grown in the greenhouse experiment.

| **Nutrient level** | **Competitor Identity** | **JA** | **Root to shoot ratio** | |
| --- | --- | --- | --- | --- |
|  |  |  | **Ratio** | **se** |
| Low | Centaurea | - | 1.6313 | 0.1693 |
|  | Festuca | - | 1.5127 | 0.2395 |
|  | Centaurea | + | 1.5346 | 0.1597 |
|  | Festuca | + | 1.7513 | 0.2395 |
|  |  |  |  |  |
| High | Centaurea | - | 0.7569 | 0.0591 |
|  | Festuca | - | 0.6657 | 0.0091 |
|  | Centaurea | - | 0.7585 | 0.0688 |
|  | Festuca | + | 0.5534 | 0.0910 |
