# Supplementary material for: Plant neighbor identity influences plant biochemistry and physiology related to defense
Source: BMC Plant Biol. 2010 Jun 17;10:115. doi: 10.1186/1471-2229-10-115 (PMC3095278; doi:10.1186/1471-2229-10-115)
Supplement: Additional file 4 — Table S4. Centaurea maculosa greenhouse experiment overall ANOVA for root to shoot ratios. Root shoot ratios were log transformed to fit normality assumptions. ANOVA F and p values are given for all main effects and interactions. [file 1471-2229-10-115-S4.DOC]

**Additional File 4 - Table S4. *Centaurea maculosa* greenhouse experiment overall ANOVA for root to shoot ratios.**

Root shoot ratios were log transformed to fit normality assumptions. ANOVA F and p values are given for all main effects and interactions.

| **(log) Root to shoot ratio** | | |
| --- | --- | --- |
| *Effect* | *F* | *p* |
| Nutrient level | 109.55 | <0.0001 |
| Neighbor | 0.38 | 0.5398 |
| Elicitation | 0.01 | 0.9060 |
| Nutrient level*Neighbor | 1.99 | 0.1613 |
| Nutrient level*Elicitation | 0.25 | 0.6187 |
| Neighbor*Elicitation | 0.95 | 0.3323 |
| Nutrient level*Neighbor*Elicitation | 1.77 | 0.1872 |
