# Supplementary material for: Plant neighbor identity influences plant biochemistry and physiology related to defense
Source: BMC Plant Biol. 2010 Jun 17;10:115. doi: 10.1186/1471-2229-10-115 (PMC3095278; doi:10.1186/1471-2229-10-115)
Supplement: Additional file 5 — Table S5. Mean, standard error, and ANOVA results for all features identified by UPLC-MS demonstrating significant ANOVA effects from relative density. Mean, standard error, and ANOVA results for all features identified by UPLC-MS demonstrating significant ANOVA effects from relative density. (H, high density, conspecific stands; L, low density, heterospecific stands). [file 1471-2229-10-115-S5.DOC]

**Additional File 5 - Table S5. Mean, standard error, and ANOVA results for all features identified by UPLC-MS demonstrating significant ANOVA effects from relative density.**

Mean, standard error, and ANOVA results for all features identified by UPLC-MS demonstrating significant ANOVA effects from relative density. (H, high density, conspecific stands; L, low density, heterospecific stands).
