# Supplementary material for: Plant neighbor identity influences plant biochemistry and physiology related to defense
Source: BMC Plant Biol. 2010 Jun 17;10:115. doi: 10.1186/1471-2229-10-115 (PMC3095278; doi:10.1186/1471-2229-10-115)

**Additional File 6 - Table S6. Soil physiochemical properties.**

Soil properties in heterospecific (L, low density of *C. maculosa*) and conspecific (H, high density of *C. maculosa*) stands. Standard deviations are noted in parenthesis for each measurement. None of the measurements were significantly different between stand type at p = 0.01.


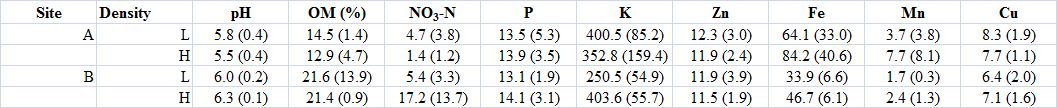

Supplement: Additional file 6 — Table S6. Soil physiochemical properties. Soil properties in heterospecific (L, low density of C. maculosa) and conspecific (H, high density of C. maculosa) stands. Standard deviations are noted in parenthesis for each measurement. None of the measurements were significantly different between stand type at p = 0.05. [file 1471-2229-10-115-S6.DOC]
